# The Ecology and Feeding Habits of the Arboreal Trap-Jawed Ant *Daceton armigerum*

**DOI:** 10.1371/journal.pone.0037683

**Published:** 2012-06-21

**Authors:** Alain Dejean, Jacques H. C. Delabie, Bruno Corbara, Fréderic Azémar, Sarah Groc, Jérôme Orivel, Maurice Leponce

**Affiliations:** 1 CNRS, Écologie des Forêts de Guyane (UMR-CNRS 8172), Campus Agronomique, Kourou, France; 2 Université de Toulouse, UPS (Ecolab), Toulouse, France; 3 U.P.A. Laboratório de Mirmecologia, Convênio UESC/CEPLAC, Itabuna, Bahia, Brazil; 4 CNRS, Laboratoire Microorganismes, Génome et Environnement (UMR-CNRS 6023), Université Blaise Pascal, Aubière, France; 5 Clermont Université, Université Blaise Pascal (LMGE), Clermont-Ferrand, France; 6 CNRS, Laboratoire d'Ecologie Fonctionnelle et Environnement (UMR-CNRS 5245), Toulouse, France; 7 Instituto de Biologia Universidade Federal de Uberlândia, Uberlândia, Minas Gerais, Brazil; 8 Biological Evaluation Section, Royal Belgian Institute of Natural Sciences, Brussels, Belgium; University of Leeds, United Kingdom

## Abstract

Here we show that *Daceton armigerum*, an arboreal myrmicine ant whose workers are equipped with hypertrophied trap-jaw mandibles, is characterized by a set of unexpected biological traits including colony size, aggressiveness, trophobiosis and hunting behavior. The size of one colony has been evaluated at ca. 952,000 individuals. Intra- and interspecific aggressiveness were tested and an equiprobable null model used to show how *D. armigerum* colonies react *vis-à-vis* other arboreal ant species with large colonies; it happens that *D. armigerum* can share trees with certain of these species. As they hunt by sight, workers occupy their hunting areas only during the daytime, but stay on chemical trails between nests at night so that the center of their home range is occupied 24 hours a day. Workers tend different Hemiptera taxa (i.e., Coccidae, Pseudococcidae, Membracidae and Aethalionidae). Through group-hunting, short-range recruitment and spread-eagling prey, workers can capture a wide range of prey (up to 94.12 times the mean weight of foraging workers).

## Introduction

The Neotropical genus *Daceton* comprises only two species [1]; *Daceton armigerum*, the most studied species, is distributed throughout northern South America. The arboreal *D. armigerum* colonies nest in the naturally hollow branches of trees or in branches hollowed out by insect larvae; they can very easily consist of up to 10,000 individuals [2], [3]. The workers have trap-jaw, hypertrophied mandibles that snap together, triggered by sensory hairs situated on the labrum and powering a killer bite [4]. The polymorphism of the worker caste is dramatic, and the size-frequency unimodal (monophasic allometry); foraging workers, themselves highly polymorphic, are larger than those from inside the colony [5], [6]. Workers are so well adapted to arboreal life that when they fall from the forest canopy they are able to glide down onto the trunk of their host tree [7].

*Daceton armigerum* workers use trail pheromones drawn from poison gland contents that remain active for more than 7 days. Trails laid with the sternal glands, relatively short-lived, serve to recruit nestmates to food patches, while secretions from the pygidial gland release attractants to food at short range (up to 15 cm) [3], [5], [8], [9]. Short-range recruitment can also be elicited through visual signals [2], [5].

Workers are visual predators that hunt diurnally; by keeping their long trap-jaw mandibles open to ca. 180° [2] they are frequently able to capture a wide range of prey, including relatively large items that they retrieve in groups of up to six ants [2], [3], [6]. During prey capture, the workers can sting the prey; their poison gland contains a mixture of pyrazines [10]. Also, carbohydrates seem limited in the diet of this species. Indeed, trophobiosis has been reported only once for workers tending coccids [11]. Yet, life for arboreal ants, particularly those species with large colonies, cannot only be based on the results of their predatory activity, so that their ability to exploit different plant-derived food sources such as extrafloral nectar and the honeydew of sap-sucking hemipterans is primordial [12]–[14].

Due to its particularity of being an arboreal species with workers having trap-jaw mandibles, we decided to study the following ecological traits of *D. armigerum*: (1) the extent of the zones occupied by the colonies, (2) the aggressiveness of the workers *vis-à-vis* competing ants, (3) their daily rhythm of activity, and, more specifically, (4) their feeding habits, including trophobiosis and predation.

## Materials and Methods

### Ethics Statement

This study was conducted according to relevant national and international guidelines. Sample collections necessary to scientific research were authorized by the French *Office National des Forêts* (*ONF*), provided that their impact upon the environment is considered negligible (see ONF-Guyane at http://www.onf.fr/guyane/@@index.html).

### Study areas

Because *D. armigerum* is quite infrequent (noted on only one out of 167 trees in the canopy; [15]), data gathering was staggered between December 1992 and July 2011 to permit us to have enough cases for a comparative study. We worked in the primary rainforests of French Guiana around the Petit Saut dam (Sinnamary; 05°03′30″N; 52°58′34.6″W); at the Paracou experimental site (05°18′N; 52°55′W); along the Voltaire River (05°03′10″N; 54°05′18″W); in a wooded area in Awala-Yalimapo (05°44.733′N; 53°56.354′W) and then along the road leading to Mana; in a gallery forest at the foot of the Montagne des Singes (05°04′20″N; 52°40′49″W); at Kaw Mountain (04°43′60″N; 52°17′60″W); and on the forested plateau at the Nouragues Research Station (04°05′20″N; 52°40′28″W). We also worked twice in Kourou (November 2005–August 2006 and April 2008–July 2009) where we transported branches containing large parts of colonies (evaluated at more than 3,000 workers, brood, plus several queens in both cases) from tall, downed trees in the forest to a home garden. The transported branches were tied to those of a small tree where workers were free to forage, permitting easy observations that began 8 days after installation.

### Size of the *D. armigerum* colonies and extent of the zones they occupied

The size of the colonies was estimated from three fallen trees sheltering a part of a *D. armigerum* colony. In the field, we firstly sawed off the zones of the trunk (*Cecropia sciadophylla* at Petit Saut) and/or branches where the exit hole to one of the colony chambers was visible. These pieces of trunk and branches were then completely opened (see picture in [3]), and the queens, workers and brood placed into a plastic container whose upper walls were coated with Fluon ® to prevent the workers from climbing out. After counting them, we released the ants or placed them with those from the same colonies installed in the garden in Kourou. The shape of the *C. sciadophylla* chambers was cylindrical (up to 9.5 cm in diameter for 58 cm in high; 1730 cm^3^) but very irregular in the other cases (cylindrical: 3–8 cm in diameter, for lengths of up to 45 cm; or flattened: height×width×length of up to 2 cm×6 cm×40 cm). We also noted the number of individuals gathered from 75 small cavities (cylindrical, less than 1 cm in diameter, 7–16 cm in length) hollowed out by borers at the ends of the branches of these trees. Yet, it is very difficult to make an estimation here as the number of these cavities varies greatly between areas on the same tree and from one tree or tree species to another.

The number of individuals was then extrapolated from the number of exit holes noted on the large branches and trunks of the three fallen trees and then multiplied by the number of large trees. For small trees, we counted the number of exit holes on 14 trees. The final estimation corresponded to the formula: (mean number of individuals per chamber * mean number of chambers per large tree* number of large trees)+(mean number of individuals per chamber * mean number of chambers per small tree* number of small trees).

Because the vegetation is low, the canopy is partly visible, enabling us to pinpoint conspicuous *D. armigerum* foragers and thus evaluate the extent of the areas occupied by the colonies. This was especially true for the sites along the Voltaire River and along the beach in Awala-Yalimapo. In Paracou and the Nouragues field stations we climbed trees to look for colonies, while in the other cases a part of the colony nested in a fallen tree. After having noted the presence of workers in an area, we baited the trees using honey, tinned tuna and insects caught at a light trap and frozen for safe-keeping. The baits were deposited at three different heights (i.e., 1.5 to 2 m) on the bark of the tree trunks and/or low branches. We then noted the number and distribution of trees on which we observed workers.

In Awala-Yalimapo, we worked in a forest fragment of ca. 5 ha where we had already noted the presence of *D. armigerum* workers. To have an idea of the extent of the area occupied by the *D. armigerum* colony, we firstly baited all of the tree trunks as indicated above. We then searched over a 30 m×100 m transect in order to assess the distribution of the different ant species likely to compete or share trees with *D. armigerum*.

### Relationships with sympatric arboreal ant species

#### Bioassays highlighting intra- and interspecific aggressiveness

Intraspecific confrontations were conducted between foraging individuals gathered from (1) two different locations on the same tree (same tree tests), (2) trees from different extremities of the same patches (intra-patch tests), and (3) trees from different patches (ca. 30 to 250 Km from each other; inter-patch tests). We transported workers in plastic containers whose upper walls were coated with Fluon ®. For longer transfers, we placed pieces of wood from the workers' host trees into the containers, plus two small test-tubes containing cotton imbibed with water and honey, respectively. Due to the difficulty of having at least two colonies available at the same time, the inter-patch tests were conducted using the *D. armigerum* colonies from the garden in Kourou. Interspecific confrontations were conducted using *Azteca* sp. *pittieri* complex, *Crematogaster carinata* and *Dolichoderus bispinosus* workers.

During the bioassays, we placed the worker to be tested on the host tree branches of a resident colony less than 5 cm from a nest entrance guarded by three to five *D. armigerum* workers. We scored the behaviors of the introduced and resident ants for the first encounter as follows. (1) accept (the introduced worker moves easily among the residents that completely ignore it; in an intraspecific confrontation it can even enter the colony chamber); (2) inspect (the introduced worker is antennated during more than 30 s and then tolerated); (3) retreat (the introduced worker moves quickly to avoid contact with the residents; it can also immediately jump from the branch after perceiving their presence); (4) show aggression (the introduced worker is seized by an appendage but later released); and (5) fight (prolonged biting, reciprocal fighting, biting by several residents, the use of defensive compounds).

We conducted 25 replicates for each situation and compared behaviors using the *Chi*-square test (Past software; [16]) followed by a sequential Bonferroni correction for multiple comparisons [17].

#### Field studies

A detailed survey was conducted over a 30 m×100 m transect (131 trees) in Awala-Yalimapo. Our aim was to rapidly assess the distribution of the dominant arboreal ants over a transect (not to conduct an exhaustive inventory of the arboreal ant assemblage). Each tree (including ca. 6-m-tall individuals growing along the beach) was baited as previously indicated. The ants were gathered and preserved in 70% ethanol for later identification to species or morphospecies and voucher specimens were deposited in the *Laboratório de Mirmecologia*, Cocoa Research Centre CEPEC/CEPLAC (Ilhéus, Bahia, Brazil).

To study the distribution of colonies of sympatric ant species, global trends in species associations were investigated using a fixed-equiprobable null model and the C-score co-occurrence index available in the EcoSim software [18]. The fixed-equiprobable algorithm maintains the species occurrence frequencies and considers all sites (trees) equiprobable [19]. The C-score index used in combination with the fixed-equiprobable algorithm has generally good statistical properties and is not prone to false positives [19]. Specific associations between the most common species likely to be dominant or co-dominant were tested using *Chi*-square tests (Yates' correction). Yet, we must keep in mind that null model co-occurrence analyses alone do not necessarily mean that competition is the structuring mechanism [20].

During the studies of the daily rhythm of activity and predation, we noted what reactions *D. armigerum* workers had *vis-à-vis* those from different species sharing their trees and, reciprocally, the behavior of the latter.

### Daily rhythm of activity

The daily rhythm of activity of the workers was firstly studied for the large parts of a colony transported to a garden in Kourou. We counted the workers entering and leaving their nests during 10 minutes each hour during several series of observations spread over 30 days, permitting us to conduct 6 to 26 replicates for each hour of the nycthemeron and to obtain means (±SE). The same was done in the field only for foraging workers from the colony situated at Base Vie that hunted daily in an easily observable area (the observations were spread over 11 days; 4 to 11 replicates).

### Trophobiosis

Each time we found *D. armigerum* foraging in an easily observable area we noted what kind of hemipterans they were tending. Also, in the garden in Kourou, we used a branch to interconnect the tree where there was a part of a colony to a *Croton* (Euphorbiaceae) on which *Camponotus* sp. tended coccids and pseudococcids. We then verified if *D. armigerum* foragers explored this tree and eventually exploited these hemipterans (this was conducted twice with parts of two different colonies).

### Prey captured by the workers and prey capture behavior

During the different studies conducted in the field we noted what kind of prey were retrieved by the workers. Using a quartz crystal microbalance, we weighed some of them (taken from the workers retrieving them), and weighed 30 ambushing workers to calculate the prey-predator weight ratio.

We studied predatory behavior in the field as a preliminary study, permitting us to note that ambushing workers reacted when an insect lands within a radius of up to 4.5 cm from them. We therefore worked in natural conditions at Base Vie by attracting flies using dead fish impaled on the end of sharpened poles and positioned 40 cm below areas where *D. armigerum* workers usually ambushed. The fish were renewed daily. After 3 days, the number of ambushing workers had increased in the area, permitting the easy analysis of their predatory behavior. We selected two size classes of flies (ca. 0.6-cm-long and ca. 1.2-cm-long individuals) and conducted the analysis (n = 30 cases) each time a fly landed within a radius of up to 4.5 cm from the head of an ambushing worker.

In the garden in Kourou, we connected the branches containing parts of nests to a wooden table using a 4-cm-wide board. We allowed the workers one week to acclimate themselves to the local environment (see the same method in [21], [22]). We then analyzed the capture of 30 ca. 2.2-cm-long grasshoppers (Tettigonidae). Using forceps, we dropped them less than 3 cm from ambushing *D. armigerum* workers. The day before each series of tests, we did not provide that colony with prey.

The behavioral sequences were recorded through direct observation. Two successive observational periods were separated by at least 1 hour. A full repertoire of behavioral sequences was first established during preliminary experiments. Referring to this complete list, we recorded each behavioral act performed and the parts of the prey body seized and those stung by the ants. We then built a flow diagram where the transition frequencies between behavioral acts were calculated based on the overall number of transitions between each individual behavioral act (see [22], [23]).

## Results

### Extent of the zones occupied by *D. armigerum* colonies and size of the colonies

Numerous field studies conducted regularly between 1992 and 2011 permitted us to note the presence of only 15 *D. armigerum* colonies and to study the extent to which nine of them had spread (Table 1). For the two largest colonies, this corresponded to 0.3 ha along the Voltaire River (more than 300 trees of different sizes, including ca. 30-m-tall individuals), and 2 ha along the beach in Awala-Yalimapo (227 trees; Table 1).

**Table 1:** Size of the territories of nine *Daceton armigerum* colonies and the estimated number of individuals.

	Geographical areas	No. of large trees (20–45 m)	No. of small trees (6–15 m)	* Size of the territory	** No. of individuals	Ant species noted on the territory of the *Daceton armigerum* colonies
1	Voltaire River	96	>200	0.300 ha	952000	*Crematogaster carinata*, *Camponotus* spp., *Cephalotes* spp.
2	Awala-Yalimapo	38	189	<2.000 ha	451520	*Azteca* spp., *Crematogaster* spp., *Camponotus* spp., *Cephalotes* spp., *Pseudomyrmex* spp., *Pachycondyla villosa* (see details in Appendix S1)
3	Paracou	1	0	0.002 ha	8500	*Azteca jelskii*
4	Nouragues	2	0	0.008 ha	17000	*Azteca instabilis*; *Crematogaster carinata*
5	Kaw mountain	7	4	0.006 ha	62220	*Camponotus balsami*
6	Petit Saut (*Base vie*)	12	6	0.200 ha	106080	*Crematogaster limata*
7	Petit Saut (PK90)	1§	0	0.001 ha	943	*Crematogaster* sp.
8	Between Yalimapo and Mana	7	0	0.009 ha	59500	*Crematogaster carinata*, *Cephalotes minutus*
9	*Montagne des singes*	4	0	0.004 ha	34000	*Crematogaster* sp., *Cephalotes minutus*

Information on the ant species sharing their territories is provided.

* Surface area of the territory projected to the ground (in hectares);

** the estimation of the number of individuals was calculated (1) from the mean number of individuals per chamber opened (ca. 340, see text), (2) the mean number of entrances noted on large, fallen trees (39, 21 and 17; resulting in a mean of 8726 *D. armigerum* individuals per large tree, rounded down to 8500), and the mean number of entrances noted on 14 easily accessible small trees (2.21 chambers per small tree, rounded down to 2, resulting in a mean of 680 individuals per small tree);

§ a 40-m-tall isolated dead tree sheltering a large *Clusia grandifolia* hemi-epiphyte (Clusiaceae) plus large shoots of the epiphytic Araceae *Philodendron solimoesense*.

By thoroughly opening 25 “large” chambers from three colonies, we noted the presence of one to five queen(s) per chamber, 24 to 467 workers, 34 to 194 larvae and pupae, and numerous eggs plus first instar larvae (means ± SE: 2.56±0.23; 258.72±17.63; 80.36±8.11; for queens, workers and larvae plus nymphs, respectively). The mean number of individuals per chamber was 341.64±24.86 (eggs and first instar larvae not included), rounded down to 340 for the estimations presented in Table 1 where the largest colony contained ca. 952,000 individuals. This result is under-evaluated as we noted five to 22 workers (10.96±0.53) per hollow branch extremity corresponding to “small” chambers; only once were larvae present. The colony nesting between the roots of a *Philodendron solimoesense* and the upper part of an isolated 30-m-tall dead tree, with only four queens and 939 workers plus larvae and nymphs, was small compared to the others (Table 1).

The colonies likely contain multiple egg-laying queens as none of the queens observed had wing stubs. Indeed, in many ant species, non-mated females that remain or return to their nests lose their wings piece by piece, leaving stubs. On the contrary, after the nuptial flight, the queens use their hind legs to tear their wings off. This is possible due to the presence of a line of predetermined weakness situated at the base of wings [24] and results in a neat tear usually considered an indication of having mated.

### Relationships with sympatric arboreal ant species

#### Bioassays highlighting intra- and interspecific aggressiveness

**Figure 1:**
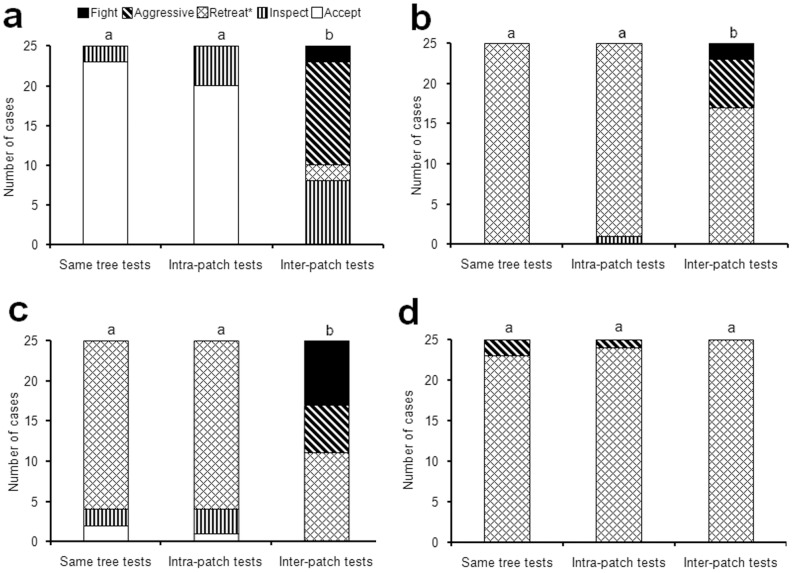
Different levels of aggressiveness noted on the part of *Daceton armigerum* guards towards workers. They originated from the same tree (same tree tests), a different tree belonging to the same patch thought to belong to the territory of the same *D. armigerum* colony (intra-patch tests), and two different patches (inter-patch tests). The introduced worker is another *Daceton armigerum* (a), an *Azteca* sp. *pittieri* complex (b), a *Crematogaster carinata* (c) and a *Dolichoderus bispinosus* (d). Statistical comparisons: *Chi*-square tests and sequential Bonferroni correction; different letters above the plots indicate significant differences (P<0.001 for a and c; P<0.05 for b; N = 25 in all cases).

During confrontation tests, *D. armigerum* guards tolerated conspecific workers gathered from their own tree as well as those from the same patch (non-significant differences; Fig. 1a), while conspecific workers from another patch were frequently attacked (significant differences with both previous cases; Fig. 1a). It is therefore likely that workers from the same patch belong to the same colony; we never observed two colonies controlling different parts of the same patch.

During interspecific confrontations, the introduced workers generally tried to retreat. Yet, as in the previous case, workers gathered from another patch were attacked each time they did not retreat fast enough (significant differences with individuals from the same tree and from the same patch; Fig. 1b,c). *Dolichoderus bispinosus* workers mostly retreated (non-significant differences between the situations; Fig. 1d). The absence of aggressiveness noted here is likely due to the fact that introduced *Dol. bispinosus* workers retreated quickly, while the *D. armigerum* guards, for their part, did not try to strike them, resulting in a form of reciprocal avoidance (*Dol. bispinosus* workers held with forceps in front of a nest entrance were always struck, with the *D. armigerum* moving backward just after striking; 15 tests).

#### Distribution of colonies of sympatric ant species in Awala-Yalimapo

The null model analysis indicated that in general species co-occurred less frequently than expected by chance (P<0.001) suggesting the existence of an ant mosaic [25]. More specifically, we found negative associations in the co-occurrences between *D. armigerum* and *Dol. bispinosus* in addition to other cases involving the latter species, *Cr. brasiliensis*, *Azteca* sp. and *Camponotus fastigatus* (Table 2). However, three positive associations were found involving *Azteca* sp. and *Ca. fastigatus*, *Cr. brasiliensis* and *Ca. trapezoideus*, and *D. armigerum* and *Cephalotes clypeatus*; workers of the latter species, whose colonies are relatively small, are very similar in shape and color to small *D. armigerum* foraging workers (which is kind of Batesian mimicry).

**Table 2:** Associations between the most frequent species (relative frequency >5%) from the Awala-Yalimapo transect.

	Relative frequency	Species	1	2	3	4	5	6	7
1	49%	*Daceton armigerum*							
2	34%	*Azteca* sp. *pittieri* complex	0						
3	19%	*Camponotus fastigatus*	0	+					
4	17%	*Camponotus trapezoideus*	0	0	(−)				
5	15%	*Crematogaster brasiliensis*	0	(−)	(−)	+			
6	11%	*Dolichoderus bispinosus*	(−)	(−)	0	0	0		
7	8%	*Cephalotes clypeatus*	+	0	0	0	0	0	
8	7%	*Crematogaster carinata*	0	0	0	0	0	0	0

The associations were sorted by decreasing rank of occurrence and tested using *Chi*-square tests (1 df, Yates' correction). Symbols indicate the nature of the association: +: positive, (−) negative, 0: not significant. Among the species noted at large densities on numerous trees, we always found situations of co-dominance (*Crematogaster brasiliensis* and *Dolichoderus bispinosus*, the most territorial species in the area, can truly share trees; i.e., workers use the same branches).

#### Reactions vis-à-vis *Crematogaster limata* and *Azteca* sp

We noted that during the daytime some *D. armigerum* workers, mandibles open, remained immobile, their body perpendicular to the trails they shared with other ant species. While following trails, *Cr. limata* workers deviated from their path by 3–4 cm each time they passed in front of the immobile *D. armigerum* workers. Yet, the latter approached very swiftly and struck *Cr. limata* 17 times out of 103 encounters noted (16.5%). The *Cr. limata* were projected from the supporting branch (after striking, the *D. armigerum* worker immediately opened its mandibles again), whereas when they were retrieving a piece of prey the strike killed them (27 cases out of 47 encounters; 57.45%) and the *D. armigerum* robbed their piece of prey. In Awala-Yalimapo, we noted 150 similar encounters between *Azteca* sp. and *D. armigerum* workers. The latter also attacked but never hit the *Azteca* that then fled. When the *D. armigerum* workers were spread-eagling or retrieving a prey, the *Azteca* frequently tried to rob it, seizing a prey's appendage and pulling backward (Fig. S2a–c). Each time, one of the *D. armigerum* workers left the prey and approached the *Azteca* that immediately fled (Fig. S2d). The *D. armigerum* workers never struck the *Azteca* (62 observations), even those that were within reach.

### Daily rhythm of activity

**Figure 2:**
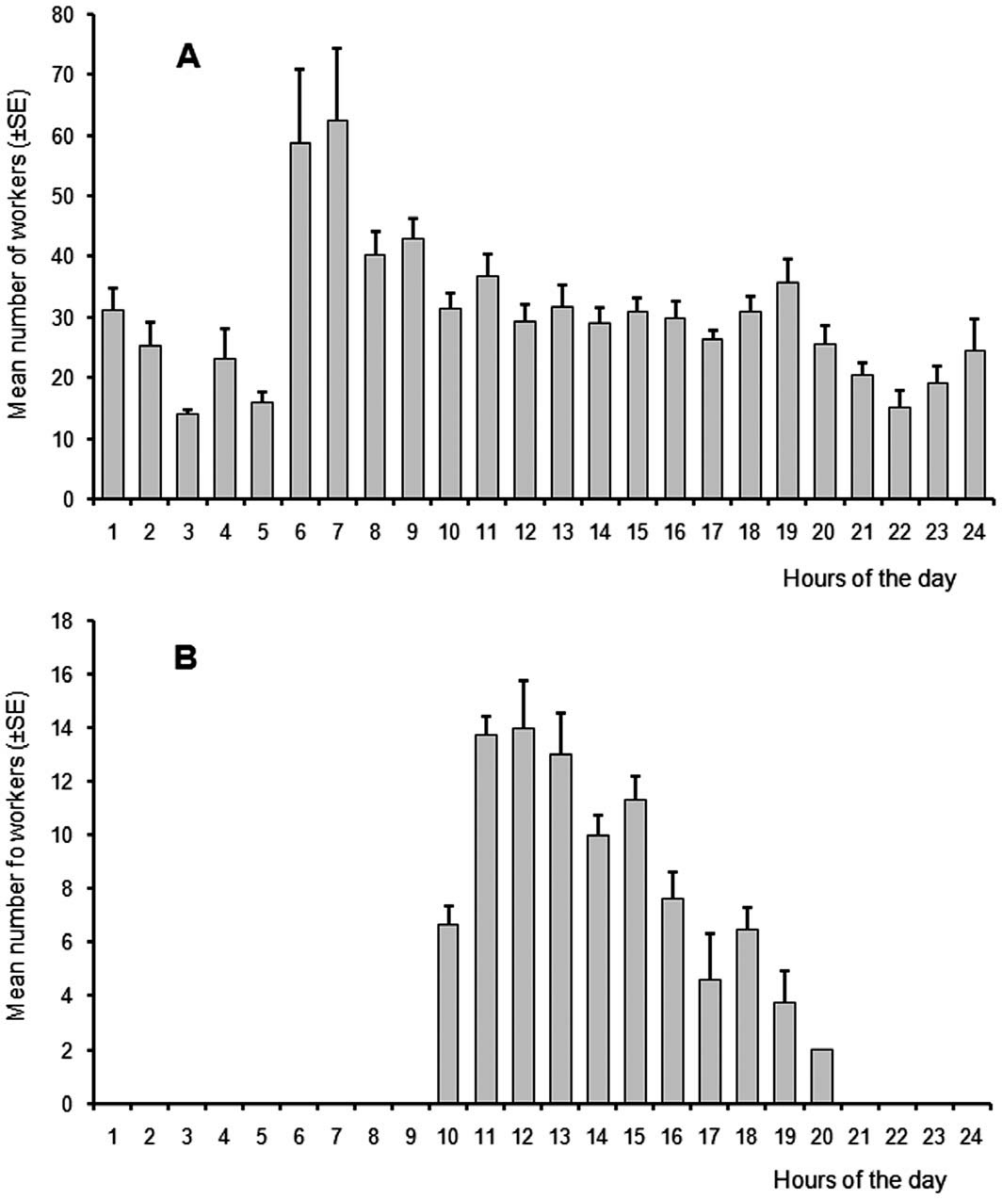
Rhythm of activity of *Daceton armigerum* workers. A. The workers were noted entering or leaving their nests (the study was conducted in a garden in Kourou over the entire nycthemeron). B. Activity in the foraging areas (the study was conducted in the field).

*Daceton armigerum* workers were active outside their nests 24 hours a day (Fig. 2A). During the night and particularly between 6:00 and 8:00 a.m., we noted individuals moving between the different chambers. Workers transported brood and sometimes callow workers (which are light yellow), while some other, older workers and queens moved on their own. However, outside these trails, that is, in the foraging areas, the rhythm of activity of the workers was typically diurnal (Fig. 2B).

### Trophobiosis

Each time we had access to the foliage of the *D. armigerum* host trees (nine colonies), we noted the ants tending Coccidae. We also noted three cases of trophobiosis with Membracidae and Aethalionidae. In the garden in Kourou, after we had connected the tree on which we had installed a part of a colony to a *Croton* where *Camponotus* sp. tended coccids and pseudococcids, *D. armigerum* scouts recruited nestmates that in turn tended the coccids and pseudococcids. After 1 week, the two ant species shared the *Croton* as well as the coccids and pseudococcids: *D. armigerum* during the daytime and *Camponotus* sp. at night.

### Prey choice and prey capture behavior

Hunting *D. armigerum* workers are able to capture a wide range of arthropods, including relatively large items, the largest being a 4.5-cm-long locust weighing 1.6 g, or 94.12 times the weight of an ambushing worker (Table S1).

**Figure 3:**
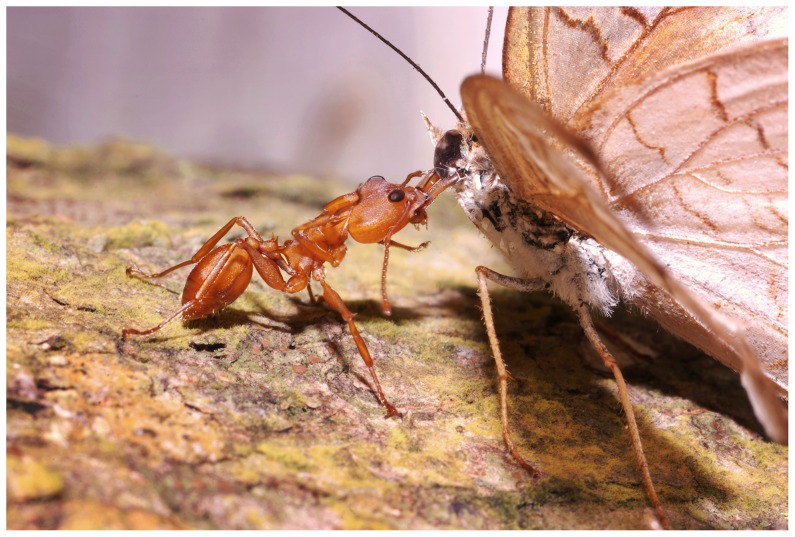
An ambushing *Daceton armigerum* worker that just seized a pierid butterfly after striking it on the head with its long mandibles. This numbed the butterfly at first, but it later struggled and was then spread-eagled by six recruited workers. One can note the well-developed claws on the pretarsa, at the extremities of the worker's legs, permitting it to get a good grip on the bark of the host tree.

*Daceton armigerum* workers ambush mostly on their host-tree branches. The distance between a successful worker and its nearest neighbor was 8 to 18 cm (mean±SE; 11.73±0.58 cm; 30 cases). Ambushing workers detect prey by sight and can begin their lightning approach before the prey has landed, striking them immediately. Many prey were seized by the head (significant difference with a random seizure; Figs. 3, S3, S4, S5). The strike permitted the ants to immobilize 100% of the flies and 80% of the ca. 2.2-cm-long grasshoppers (the remaining 20% were able to struggle but were held onto by the attacking workers). Some flies were retrieved just after being seized by the attacking worker (36.7% and 6.7% for small and large flies, respectively). Otherwise, the prey were spread-eagled thanks to the rapid arrival of workers recruited at short range (Figs. S3, S4, S5). Indeed, 44 out of the 60 recruited individuals we observed firstly touched the tip of the gaster of the recruiting individual. If a new individual arriving by chance near the tip of the first attacking worker's gaster can be evaluated at one case out of six, the difference between observed and theoretical cases is significant (44 cases out of 60 *versus* 10 cases out of 60; Fisher's exact-test: P<0.0001). To spread-eagle the prey, which can take up to 1 hour for grasshoppers, each worker seizes a prey appendage (which is facilitated by the shape of the extremities of the worker's mandibles; see Fig. S6) or a part of the body and pulls backward.

The prey can be stung by the first attacking worker (all cases for flies retrieved by a single worker) or by recruited nestmates during spread-eagling. In the latter case, the workers bit the prey's leg, and then bent their gaster under their alitrunk so that their stinger reached the intersegmental membrane separating the coxa of the seized leg and the prey thorax (this area is close to the neural chain, facilitating paralysis; see Figs. S4c, d). Long-range recruitment (some workers, firstly recruited at short-range, returned to the nest leaving a trail to recruit new nestmates) occurred only for 2.2-cm-long grasshoppers (Fig. S3) with the number of recruited workers reaching up to 20 individuals (see also Fig. S5 for spread-eagled locusts). Among the recruited workers, certain did not participate in spread-eagling the prey but rather licked the fluids that leaked out as the prey were stretched. Some prey were even partially torn apart during spread-eagling (Fig. S3).

## Discussion

The size of *D. armigerum* colonies and extent of their range can be very large, something confirmed through bioassays on the workers' intraspecific aggressiveness. The colonies, which are polygynic (multiple queens) and polydomous (multiple nests), can reach to ca. 952,000 individuals (so, even much more than the 10,000 workers suggested by Wilson [2]). We can compare them to those of the well studied arboreal weaver ant *Oecophylla* estimated at ca. 500,000 workers [24]. Furthermore, only *D. armigerum* workers shelter in the small chambers situated at the end of branches on host trees which is reminiscent of the “barracks” leaf nests built by *Oecophylla* beyond the limits of their territories and containing only old workers [24].

Intra- and interspecific aggressiveness were also shown for *D. armigerum*. More specifically, *D. armigerum* does not share trees with *Dol. bispinosus* at Awala-Yalimapo (Tables 2 and S1; Fig. S1). It is indeed known that *D. armigerum* can compete with aggressive *Azteca* plant-ants to nest in myrmecophitic *Cecropia obtusa* [26]. Yet, as already noted for other Neotropical ant species (see [15]), *D. armigerum* frequently shares trees with large colonies of other arboreal ants. These situations are not entirely peaceful as *D. armigerum* workers frequently kill the *Crematogaster* individuals with which they even share trails and rob their prey (cleptobiosis). *Azteca* sp. workers likely benefit from having defensive compounds as, when they are trying to rob prey from *D. armigerum* workers, they are never struck even when within reach; they always retreated if chased (Fig. S2).

*Daceton armigerum* workers are active around the clock along the paths interconnecting the nest chambers and they use their poison gland to lay long-lasting (more than 7 days) trails to interconnect the chambers of their nests [6], [9]. Finally, as already reported [2], [6], *D. armigerum* workers only hunt during the daytime. All of these behaviors are reminiscent of those noted for *Oecophylla longinoda* [24], [27], [28].

Trophobiosis, already reported once [11], seems frequent, but can only be confirmed if observers have access to the uppermost part of the canopy (e.g., a fallen tree; or through the use of canopy access methods) or find a colony restricted to low vegetation. Also, *D. armigerum* workers prevented *Camponotus* sp. from attending hemipterans during the daytime and so were dominant at this permanent food resource, but with respect to their own rhythm of activity. This occurred without fighting as noted for African arboreal ants [29], [30]. Trophobiosis in ants is associated with a modified proventriculus that enables workers to effectively harvest and retrieve sugar-rich honeydew [31], [32] that fuels their energy-costly foraging and territorial behavior [13], [14], [31]–[33]. In addition, because the probability of capturing prey is relatively limited in tree foliage, the workers have a thin cuticle and non-proteinaceous venom so that their need for Nitrogen is lower [31]. Yet, this is not the case for *D. armigerum* whose workers have a thick cuticle and trap-jaw mandibles [4], [24]. Moreover, their venom, although non-proteinaceous, is composed of pyrazines that contains two atoms of Nitrogen [10]. These traits are likely possible thanks to the skill of the workers at capturing prey.

The *D. armigerum* predatory behavior, based on spread-eagling prey while several workers hunt visually or within reach of the pheromones responsible for short-range recruitment, was noted for different arboreal ants having large colonies [21], [22], [27], [34], [35]. Note that group hunting accompanied by short-range recruitment is considered to be a more ‘evolved’ strategy than solitary hunting because it implies cooperation between workers and enables a species to exploit a greater range of prey sizes or food sources [36]. Also, even relatively small prey can be spread-eagled (see Fig. S7) as is the case for *Oecophylla* that capture and then singly retrieve only very small prey [27], [34]. The main difference with other arboreal ant species is based on the morphology of the mandibles of the *Daceton* workers that function like trap-jaws [4]. It is likely that a strike, which can numb even relatively large insects, is at the basis of the numerous successful captures we noted during our surveys as the prey were numbed enough to permit nestmates to be recruited at short range even if in certain cases the nearest nestmate was up to 18 cm away. Indeed, spread-eagling prey requires an efficacious short-range recruitment which here is based on visual signals [2], [6] plus secretions from the pygidial gland [5], [6]. This explains why, like for *Pheidole* [37], numerous workers recruited at short-range first antennated the tip of the gaster of the recruiting worker. Long-range recruitment frequently occurs in arboreal ants during the capture of a large prey [24], [33]; *D. armigerum* workers lay their recruitment trails using the sternal gland [9].

If compared to other predatory arboreal ants hunting in a group, the prey-predator weight ratio of up to 1∶94.12 for *D. armigerum* (this study) is slightly superior to what is typically recorded for *Oecophylla* (up to 1∶50); however, the latter can retrieve exceptionally large prey (ratio of 1∶580; [38]). Yet, these values are far lower than those noted for *Azteca andreae* with a ratio of 1∶13,350 possible thanks to a much more elaborate group hunting strategy [39].

In conclusion, *D. armigerum* combines several traits generally noted in some other arboreal ants i.e., populous colonies, large and/or polydomous nests, intra- and interspecific aggressiveness, trophobiosis, and capturing prey by spread-eagling them. So, this species likely plays an important role in structuring the Neotropical arboreal ant community.

## Supporting Information

Appendix S1 Ant species along the transect located at Awala-Yalimapo.
(DOC)

Table S1 Different arthropods naturally captured by ambushing *Daceton armigerum* workers.
(DOC)

Figure S1 Distribution of the principal arboreal ant species noted along the transect at Awala-Yalimapo.
(TIF)

Figure S2 *Azteca* sp. workers trying to rob a wasp captured by *Daceton armigerum* workers.
(TIF)

Figure S3 Behavioral sequences during predation by ambushing *Daceton armigerum* workers when prey land (flies) or are dropped (grasshoppers) less than 3 cm from them.
(TIF)

Figure S4 During the attacks ambushing workers face the prey and strike them on the head. This likely numbs the prey until nestmates can be recruited at short range.
(TIF)

Figure S5 Spread-eagling the prey.
(TIF)

Figure S6 Illustration that the shape of the tip of the *Daceton armigerum* mandibles permits them to easily seize prey appendages.
(TIF)

Figure S7 Spread-eagling flies or relatively small prey.
(TIF)
