# Supplementary material for: The Ecology and Feeding Habits of the Arboreal Trap-Jawed Ant Daceton armigerum
Source: PLoS One. 2012 Jun 21;7(5):e37683. doi: 10.1371/journal.pone.0037683 (PMC3380855; doi:10.1371/journal.pone.0037683)
Supplement: Appendix S1 — Ant species along the transect located at Awala-Yalimapo. (DOC) [file pone.0037683.s001.doc]

**Supporting Information**

***Daceton armigerum*: an uncommon but territorially-dominant arboreal ant species in Neotropical rainforest canopies**

**Appendix S1.** Ant species along the transect conducted at Awala-Yalimapo (131 trees; see Fig. S1).

| **Trees** | Ant species noted on each tree |
| --- | --- |
| **1** | *Azteca* sp. *pittieri* complex (hereafter: *Azteca* sp.) *+ Pheidole* sp. *fallax group* |
| **2** | *Azteca* sp. |
| **3** | *Crematogaster* sp.1 |
| **4** | *Dolichoderus bispinosus* |
| **5** | *Dolichoderus bispinosus* |
| **6** | *Dolichoderus bispinosus* |
| **7** | *Azteca* sp. *+ Camponotus renggeri + Pheidole* sp. *fallax group* |
| **8** | *Azteca* sp. *+ Camponotus renggeri + Pheidole* sp. *fallax group* |
| **9** | ***Daceton armigerum*** + *Camponotus renggeri* |
| **10** | ***Daceton armigerum*** + *Pseudomyrmex tenuissimus* |
| **11** | ***Daceton armigerum*** |
| **12** | *Crematogaster* sp.1 |
| **13** | *Dolichoderus bispinosus* |
| **14** | *Dolichoderus bispinosus* |
| **15** | *Dolichoderus bispinosus* |
| **16** | *Dolichoderus bispinosus* |
| **17** | *Azteca* sp. *+ Pheidole* sp. *fallax group* |
| **18** | *Dolichoderus bispinosus* |
| **19** | *Dolichoderus bispinosus* |
| **20** | *Camponotus crassus* |
| **21** | *Crematogaster* sp.1 |
| **22** | ***Daceton armigerum*** |
| **23** | ***Daceton armigerum*** *+ Camponotus renggeri* + *Cephalotes clypeatus* |
| **24** | ***Daceton armigerum*** *+ Camponotus renggeri* |
| **25** | ***Daceton armigerum*** |
| **26** | *Dolichoderus bispinosus* + *Crematogaster* sp.1 |
| **27** | *Dolichoderus bispinosus* + *Crematogaster* sp.1 + *Cephalotes pallens* |
| **28** | *Dolichoderus bispinosus* |
| **29** | *Dolichoderus bispinosus* |
| **30** | *Dolichoderus bispinosus* |
| **31** | *Azteca* sp. *+ Camponotus fastigatus* |
| **32** | *Azteca* sp. *+ Camponotus fastigatus* |
| **33** | *Azteca* sp. *+ Camponotus fastigatus* |
| **34** | *Camponotus fastigatus* |
| **35** | *Daceton armigerum + Camponotus fastigatus* |
| **36** | *Azteca* sp. *+ Camponotus novogranadensis* |
| **37** | ***D. armig*** *+ Camponotus* fastigatus + *Paratrechina longicornis* + *Tapinoma melanocephala* |
| **38** | ***D. armigerum*** + *Camponotus fastigatus* |
| **39** | ***D. armigerum*** + *Paratrechina longicornis* + *Camponotus fastigatus* |
| **40** | ***Daceton armigerum*** |
| **41** | *Camponotus novogranadensis* |
| **42** | *Camponotus fastigatus* |
| **43** | *Azteca* sp. *+ Camponotus fastigatus* |
| **44** | *Azteca* sp. *+ Camponotus fastigatus* |
| **45** | *Azteca* sp. *+ Camponotus fastigatus* |
| **46** | *Dolichoderus bispinosus + Crematogaster brasiliensis + Cephalotes minutus* |
| **47** | *Azteca* sp. *+ Cephalotes minutus + Pachycondyla villosa* |
| **48** | ***D. armigerum*** *+ Crematogaster* *brasiliensis* + *Pachycondyla villosa* |
| **49** | *Crematogaster brasiliensis + Camponotus trapezoideus* |
| **50** | ***D. armigerum*** + *Camponotus trapezoideus* + *Cre brasiliensis* + *Pseudomyrmex gracilis* |
| **51** | ***D. armigerum*** *+ Crematogaster* brasiliensis + *Cephalotes* *clypeatus* |
| **52** | ***D. armigerum*** + *Azteca fasciata* + *Cephalotes* *clypeatus* |
| **53** | ***D. armigerum*** + *Camponotus fastigatus* + *Cephalotes clypeatus* |
| **54** | Cephalotes *clypeatus* |
| **55** | ***D. armigerum*** *+ Azteca* sp. + *Campo fastigatus* + *Cephalotes clypeatus* |
| **56** | ***D. armigerum*** *+ Crematogaster brasiliensis* |
| **57** | ***D. armigerum*** *+ Azteca* sp.*+ Camponotus fastigatus* + *Cephalotes clypeatus* |
| **58** | *Azteca* sp. *+ Camponotus fastigatus* |
| **59** | *Azteca* sp. *+ Camponotus fastigatus* |
| **60** | *Crematogaster brasiliensis* |
| **61** | *Azteca* sp. *+ Camponotus trapezoideus* |
| **62** | *Crematogaster brasiliensis + Camponotus trapezoideus* |
| **63** | *Ectatomma bruneum* (nest at the base of the tree) |
| **64** | *Crematogaster brasiliensis + Camponotus trapezoideus* |
| **65** | *Crematogaster brasiliensis* + *Camponotus trapezoideus* |
| **66** | ***D. armigerum*** + *Camponotus* *trapezoideus* |
| **67** | ***D. armigerum*** + *Cephalotes clypeatus* + *Pseudomyrmex gracilis* |
| **68** | ***D. armigerum*** + *Camponotus trapezoideus* + *Cephalotes* *clypeatus* |
| **69** | ***D. armigerum*** *+ Cre brasiliensis* + *Camponotus trapezoideus* + *Pseudomyrmex gracilis* |
| **70** | ***D. armigerum*** + *Camponotus fastigatus* + *Cephalotes clypeatus* |
| **71** | ***D. armigerum*** + *Dolichoderus lutosus* |
| **72** | ***D. armigerum*** *+ Campo novogranadensis + Dolichoderus lutosus + Oligomyrmex* sp. |
| **73** | *Crematogaster brasiliensis* + *Camponotus trapezoideus* |
| **74** | ***D. armigerum*** *+ Crematogaster brasiliensis* + *Camponotus trapezoideus* |
| **75** | ***D. armigerum*** *+ Crematogaster brasiliensis* |
| **76** | ***D. armigerum*** + *Azteca* sp. + *Cephalotes* *cordatus* |
| **77** | ***D. armigerum*** *+ Crematogaster* *brasiliensis* + *Camponotus trapezoideus* |
| **78** | *Crematogaster* *brasiliensis* + *Camponotus trapezoideus* |
| **79** | *Paratrechina longicornis* + *Tapinoma melanocephala* + *Pachycondyla villosa* |
| **80** | ***D. armigerum*** + *Azteca* sp. *+ Camponotus trapezoideus* |
| **81** | ***D. armigerum*** + *Azteca* sp. |
| **82** | ***D. armigerum*** + *Azteca* sp. + *Camponotus* *fastigatus* |
| **83** | *Camponotus* *fastigatus* + *Cephalotes cordatus* |
| **84** | ***D. armigerum*** + *Azteca* sp. *+ Camponotus fastigatus* |
| **85** | ***D. armigerum*** + *Azteca* sp. *+ Camponotus fastigatus* |
| **86** | ***D. armigerum*** + *Crematogaster carinata* |
| **87** | ***D. armigerum*** *+ Cre carinata* + *Camponotus trapezoideus* + *Pachycondyla villosa* |
| **88** | *Crematogaster carinata* |
| **89** | *Cephalotes* *minutus* + *Pseudomyrmex* *tenuissimus* + *Pseudomyrmex gracilis* |
| **90** | ***Daceton armigerum*** (very large nest) |
| **91** | ***D. armigerum*** *+ Camponotus trapezoideus* |
| **92** | ***D. armigerum*** *+ Azteca* sp. |
| **93** | ***D. armigerum*** *+ Azteca* sp. |
| **94** | *Crematogaster brasiliensis* + *Tapinoma melanocephala* |
| **95** | *Azteca* sp. *+ Pseudomyrmex tenuissimus* |
| **96** | ***D. armigerum*** *+ Camponotus rengeri* |
| **97** | ***Daceton armigerum*** |
| **98** | ***D. armigerum*** *+ Azteca* sp. *+ Camponotus trapezoideus + Pseudomyrmex gracilis* |
| **99** | *Azteca* sp. |
| **100** | *Tapinoma melanocephala* |
| **101** | ***Daceton armigerum*** |
| **102** | ***D. armigerum*** *+ Camponotus trapezoideus* |
| **103** | ***Daceton armigerum*** |
| **104** | ***Daceton armigerum*** |
| **105** | ***D. armigerum*** + *Azteca* sp. |
| **106** | ***Daceton armigerum*** |
| **107** | *Azteca* sp. |
| **108** | Azteca sp. + *Camponotus trapezoideus* + *Pachycondyla villosa* |
| **109** | ***D. armig*** *+ Cre carinata* + *Azteca* sp. + *Campo crassus* + *Cephalotes nilpiei* |
| **110** | ***D. armigerum*** *+ Crematogaster carinata* + *Camponotus* *fastigatus* |
| **111** | ***D. armigerum*** *+ Crematogaster carinata* |
| **112** | *Azteca* sp. |
| **113** | *Azteca* sp. |
| **114** | *Azteca* sp. |
| **115** | ***D. armigerum*** *+ Crematogaster carinata* |
| **116** | ***D. armigerum*** *+ Azteca* sp. *+ Camponotus fastigatus* |
| **117** | ***D. armigerum*** *+ Cremato carinata*+ *Campo fastigatus* + *Cephalotes pallens* |
| **118** | ***D. armigerum*** *+ Azteca* sp. |
| **119** | *Crematogaster carinata* |
| **120** | ***D. armigerum*** *+ Azteca* sp. |
| **121** | ***D. armigerum*** *+ Camponotus trapezoideus + Cephalotes minutus* |
| **122** | ***D. armigerum*** *+ Tapinoma melanocephala + Pseudomyrmex gracilis* |
| **123** | *Azteca* sp. *+ Camponotus trapezoideus + Cephalotes cordatus* |
| **124** | *Azteca* sp. *+ Camponotus trapezoideus + Cephalotes cordatus* |
| **125** | *Azteca* sp. *+ Camponotus trapezoideus + Cephalotes cordatus* |
| **126** | ***D. armigerum*** *+ Crematogaster brasiliensis* |
| **127** | *Camponotus crassus* |
| **128** | ***D. armigerum*** *+ Crematogaster brasiliensis* |
| **129** | ***D. armigerum*** *+ Azteca* sp. |
| **130** | *Azteca* sp. |
| **131** | *Azteca* sp. |

**Figure S1.** **Distribution of the principal arboreal ant species noted along the transect at Awala-Yalimapo**. Each number represents a tree. Note that, although it is not always visible in the figure, the tree crowns are in contact with each other, forming a canopy.

**Figure S2.** ***Azteca* sp. workers trying to rob a wasp captured by *Daceton armigerum* workers**. **a-c.** The *Azteca* sp. workers had seized a prey appendage and pulled backward. **d.** An *Azteca* sp. worker, chased by a *Daceton*, is fleeing. Although within reach, the *Daceton* never hit them.

**Figure S3.** **Behavioral sequences during predation by ambushing workers *Daceton armigerum*** when prey land (flies) or are dropped (grasshoppers) less than 3 cm from them. The percentages are calculated from the total number of cases. The different phases of predation are shown in the first column. Abdo.: abdomen. Mean weight of 30 ambushing *D. armigerum* workers: 0.017 g; ratio with small flies: 0.65; with large flies: 2.12; with grasshoppers: 14.7. Statistical comparisons. Comparison between the zone seized by the first attacking worker and a random seizure (25% seizure by the head or the abdomen, 50% by the thorax and legs); all data pooled; *Chi*-square test: Χ^2^ = 16.69; 2 df; P < 0.001.

**Figure S4a-d.** **During the attacks ambushing workers face the prey and strike them on the head**. This likely numbs the prey until nestmates can be recruited at short range. The attacking worker generally continues to bite the prey until it can be retrieved. **c-d.** The yellow arrows show workers stinging the prey, their stinger piercing the intersegmental membranes at the joint between the thorax and a leg. The first attacking workers still hold onto the prey by biting their head.

**Figure S5a-d.** **Spread-eagling the prey**. The yellow arrows indicate the workers that first attacked the prey by head-on it. **c.** The prey is partially dismembered (the abdomen is gone) as the workers pull backward. **d.** Here the abdomen is stretched to its maximum.

**Figure S6.** **Tip of the *Daceton armigerum* mandibles**. Illustration that its shape permits the workers to easily seize prey appendages.

**Figure S7.** **Spread-eagling flies, or relatively small prey**. **a.** The model: *Oecophylla longinoda* in Cameroon. **b.** *Daceton armigerum*: the spread-eagling of relatively small prey also occurs, particularly if the prey is not numbed by the first strike, as was the case here.

**Supplementary Ethics Statement**

Copy of the permits obtained for the described field studies by the French *Office National des Forêts* (*ONF*).
