# Supplementary material for: The Ecology and Feeding Habits of the Arboreal Trap-Jawed Ant Daceton armigerum
Source: PLoS One. 2012 Jun 21;7(5):e37683. doi: 10.1371/journal.pone.0037683 (PMC3380855; doi:10.1371/journal.pone.0037683)
Supplement: Table S1 — Different arthropods naturally captured by ambushing Daceton armigerum workers. (DOC) [file pone.0037683.s002.doc]

**Table S1.** Different arthropods naturally captured by *Daceton armigerum* ambushing workers. *0.017 g: mean weight of 30 ambushing *D. armigerum* workers.

|  | Arthropod prey | | No. of cases | % | Weight in grams | Ratio / 0.017 g* |
| --- | --- | --- | --- | --- | --- | --- |
| Arachnida | |  |  |  |  |  |
|  | Aranea | | 4 | 0.87 |  |  |
| Odonata | |  |  |  |  |  |
|  | Anisoptera (3.5 cm) | | 2 | 0.43 |  |  |
|  | Zygoptera (4.2 cm) | | 1 | 0.22 |  |  |
| Ephemeroptera | |  | 1 | 0.22 |  |  |
| Dictyoptera | |  |  |  |  |  |
|  | Cockroaches 1.2 cm | | 11 | 2.39 | 0.09 | 5.29 |
|  | Cockroach ca. 3 cm | | 2 | 0.43 | 1.05 | 61.76 |
| Isoptera | |  |  |  |  |  |
|  | Termite workers | | 15 | 3.26 | 0.009 | 0.53 |
|  | Winged termites (0.4 cm) | | 85 | 18.48 | 0.021 | 1.23 |
| Heteroptera | |  |  |  |  |  |
|  | Pentatomidae | | 6 | 1.30 |  |  |
|  | Reduviidae | | 3 | 0.65 |  |  |
|  | Unidentified | | 2 | 0.43 |  |  |
| Orthoptera | |  |  |  |  |  |
|  | Grillidae (2.5 cm) | | 3 | 0.65 | 0.45 | 26.47 |
|  | Tettigonidae (1.2 cm) | | 19 | 4.13 | 0.08 | 4.70 |
|  | Tettigonidae (2.5 cm) | | 18 | 3.91 | 0.27 | 15.88 |
|  | Acrididae (1.5 cm) | | 13 | 2.82 | 0.15 | 8.82 |
|  | Acrididae (2.5 cm) | | 7 | 1.52 | 0.37 | 21.76 |
|  | Acrididae (3.5 cm) | | 4 | 0.87 | 1.02 | 60.00 |
|  | Acrididae (4.5 cm) | | 1 | 0.22 | 1.60 | 94.12 |
| Lepidoptera | |  |  |  |  |  |
|  | Pieridae (2-3 cm) | | 8 | 1.74 |  |  |
|  | Nymphalidae (3 cm) | | 3 | 0.65 |  |  |
|  | Lycaenidae (2 cm) | | 1 | 0.22 |  |  |
|  | Hesperidae (2.2 cm) | | 1 | 0.22 |  |  |
|  | Noctuidae (2.5-3.5 cm) | | 9 | 1.95 |  |  |
|  | Others (3.5 cm) | | 1 | 0.22 |  |  |
|  | Caterpillar (2.5-3 cm) | | 6 | 1.30 | 0.15 | 8.82 |
| Hymenoptera | |  |  |  |  |  |
|  | *Camponotus* spp. winged queens | | 29 | 6.30 | 0.030 | 1.76 |
|  | *Crematogaster* spp. winged queens | | 32 | 6.95 | 0.018 | 1.06 |
|  | *Camponotus* spp. males | | 18 | 3.91 | 0.022 | 1.29 |
|  | Other winged ants | | 19 | 4.13 |  |  |
|  | Melliponinae; *Trigona* | | 4 | 0.87 | 0.016 | 0.94 |
|  | Vespidae, Polistinae, *Agelaia* spp. | | 9 | 1.95 | 0.033 | 1.94 |
| Diptera | |  |  |  |  |  |
|  | Muscidae (0.5 cm) | | 44 | 9.56 | 0.012 | 0.70 |
|  | Muscidae (1.2 cm) | | 37 | 8.04 | 0.017 | 1.00 |
|  | Tabanidae (2.0 cm) | | 4 | 0.87 | 0.037 | 2.17 |
|  | Tipulidae (3.4 cm) | | 3 | 0.65 | 0.013 | 0.76 |
|  | Culicidae (0.6 cm) | | 6 | 1.30 |  |  |
| Coleoptera | |  |  |  |  |  |
|  | Chrysomelidae adults (0.6 cm) | | 9 | 1.95 |  |  |
|  | Lampyridae | | 4 | 0.87 |  |  |
|  | Unidentifed | | 16 | 3.48 |  |  |
|  | Total | | 460 |  |  |  |

**Table S1.** Different arthropods naturally captured by *Daceton armigerum* ambushing workers. *0.017 g: mean weight of 30 ambushing *D. armigerum* workers.

|  | Arthropod prey | | No. of cases | % | Weight in grams | Ratio / 0.017 g* |
| --- | --- | --- | --- | --- | --- | --- |
| Arachnida | |  |  |  |  |  |
|  | Aranea | | 4 | 0.87 |  |  |
| Odonata | |  |  |  |  |  |
|  | Anisoptera (3.5 cm) | | 2 | 0.43 |  |  |
|  | Zygoptera (4.2 cm) | | 1 | 0.22 |  |  |
| Ephemeroptera | |  | 1 | 0.22 |  |  |
| Dictyoptera | |  |  |  |  |  |
|  | Cockroaches 1.2 cm | | 11 | 2.39 | 0.09 | 5.29 |
|  | Cockroach ca. 3 cm | | 2 | 0.43 | 1.05 | 61.76 |
| Isoptera | |  |  |  |  |  |
|  | Termite workers | | 15 | 3.26 | 0.009 | 0.53 |
|  | Winged termites (0.4 cm) | | 85 | 18.48 | 0.021 | 1.23 |
| Heteroptera | |  |  |  |  |  |
|  | Pentatomidae | | 6 | 1.30 |  |  |
|  | Reduviidae | | 3 | 0.65 |  |  |
|  | Unidentified | | 2 | 0.43 |  |  |
| Orthoptera | |  |  |  |  |  |
|  | Grillidae (2.5 cm) | | 3 | 0.65 | 0.45 | 26.47 |
|  | Tettigonidae (1.2 cm) | | 19 | 4.13 | 0.08 | 4.70 |
|  | Tettigonidae (2.5 cm) | | 18 | 3.91 | 0.27 | 15.88 |
|  | Acrididae (1.5 cm) | | 13 | 2.82 | 0.15 | 8.82 |
|  | Acrididae (2.5 cm) | | 7 | 1.52 | 0.37 | 21.76 |
|  | Acrididae (3.5 cm) | | 4 | 0.87 | 1.02 | 60.00 |
|  | Acrididae (4.5 cm) | | 1 | 0.22 | 1.60 | 94.12 |
| Lepidoptera | |  |  |  |  |  |
|  | Pieridae (2-3 cm) | | 8 | 1.74 |  |  |
|  | Nymphalidae (3 cm) | | 3 | 0.65 |  |  |
|  | Lycaenidae (2 cm) | | 1 | 0.22 |  |  |
|  | Hesperidae (2.2 cm) | | 1 | 0.22 |  |  |
|  | Noctuidae (2.5-3.5 cm) | | 9 | 1.95 |  |  |
|  | Others (3.5 cm) | | 1 | 0.22 |  |  |
|  | Caterpillar (2.5-3 cm) | | 6 | 1.30 | 0.15 | 8.82 |
| Hymenoptera | |  |  |  |  |  |
|  | *Camponotus* spp. winged queens | | 29 | 6.30 | 0.030 | 1.76 |
|  | *Crematogaster* spp. winged queens | | 32 | 6.95 | 0.018 | 1.06 |
|  | *Camponotus* spp. males | | 18 | 3.91 | 0.022 | 1.29 |
|  | Other winged ants | | 19 | 4.13 |  |  |
|  | Melliponinae; *Trigona* | | 4 | 0.87 | 0.016 | 0.94 |
|  | Vespidae, Polistinae, *Agelaia* spp. | | 9 | 1.95 | 0.033 | 1.94 |
| Diptera | |  |  |  |  |  |
|  | Muscidae (0.5 cm) | | 44 | 9.56 | 0.012 | 0.70 |
|  | Muscidae (1.2 cm) | | 37 | 8.04 | 0.017 | 1.00 |
|  | Tabanidae (2.0 cm) | | 4 | 0.87 | 0.037 | 2.17 |
|  | Tipulidae (3.4 cm) | | 3 | 0.65 | 0.013 | 0.76 |
|  | Culicidae (0.6 cm) | | 6 | 1.30 |  |  |
| Coleoptera | |  |  |  |  |  |
|  | Chrysomelidae adults (0.6 cm) | | 9 | 1.95 |  |  |
|  | Lampyridae | | 4 | 0.87 |  |  |
|  | Unidentifed | | 16 | 3.48 |  |  |
|  | Total | | 460 |  |  |  |
