# Supplementary figures and images for: The Ecology and Feeding Habits of the Arboreal Trap-Jawed Ant Daceton armigerum
Source: PLoS One. 2012 Jun 21;7(5):e37683. doi: 10.1371/journal.pone.0037683 (PMC3380855; doi:10.1371/journal.pone.0037683)

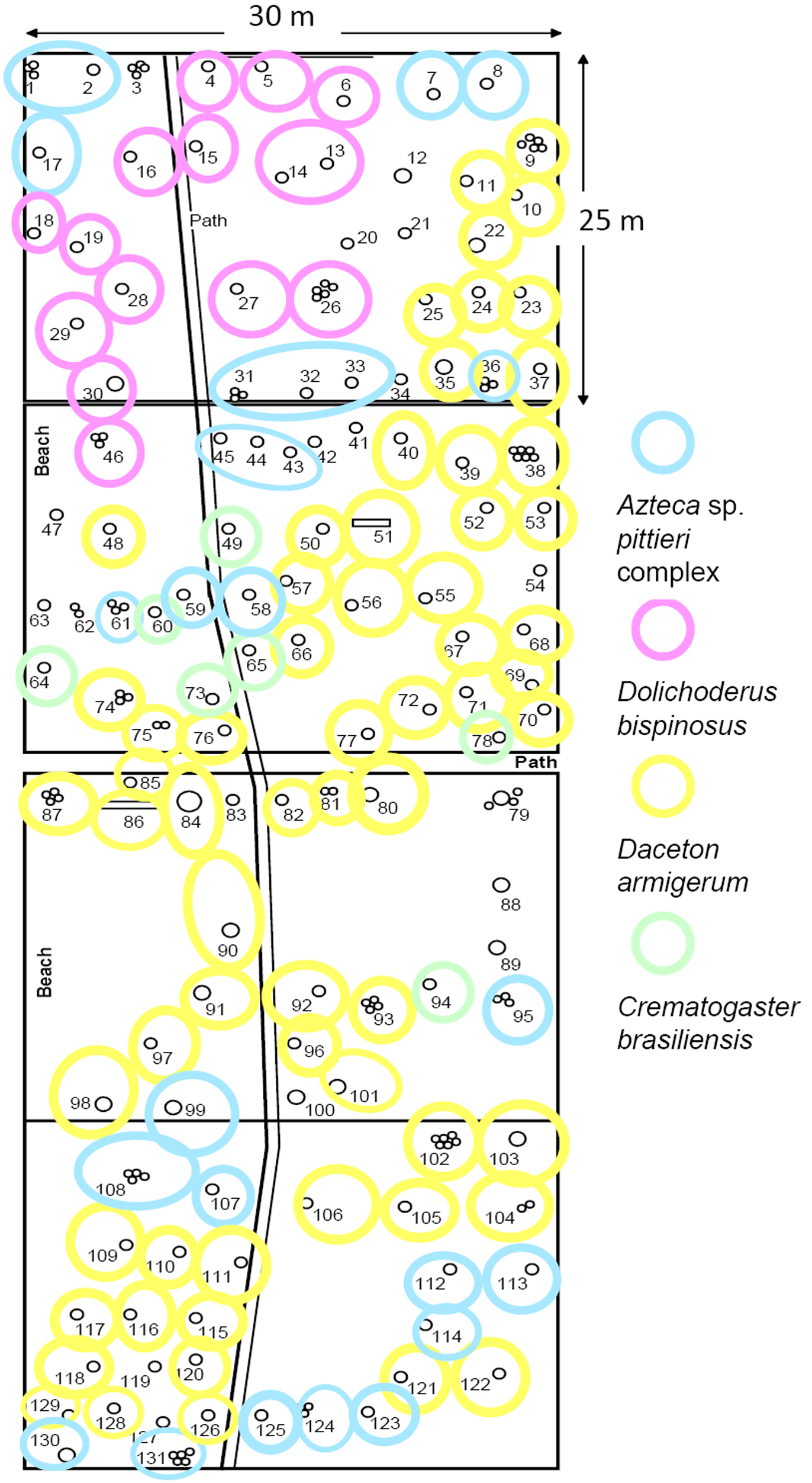

Supplement: Figure S1 — Distribution of the principal arboreal ant species noted along the transect at Awala-Yalimapo. (TIF) [file pone.0037683.s003.tif]

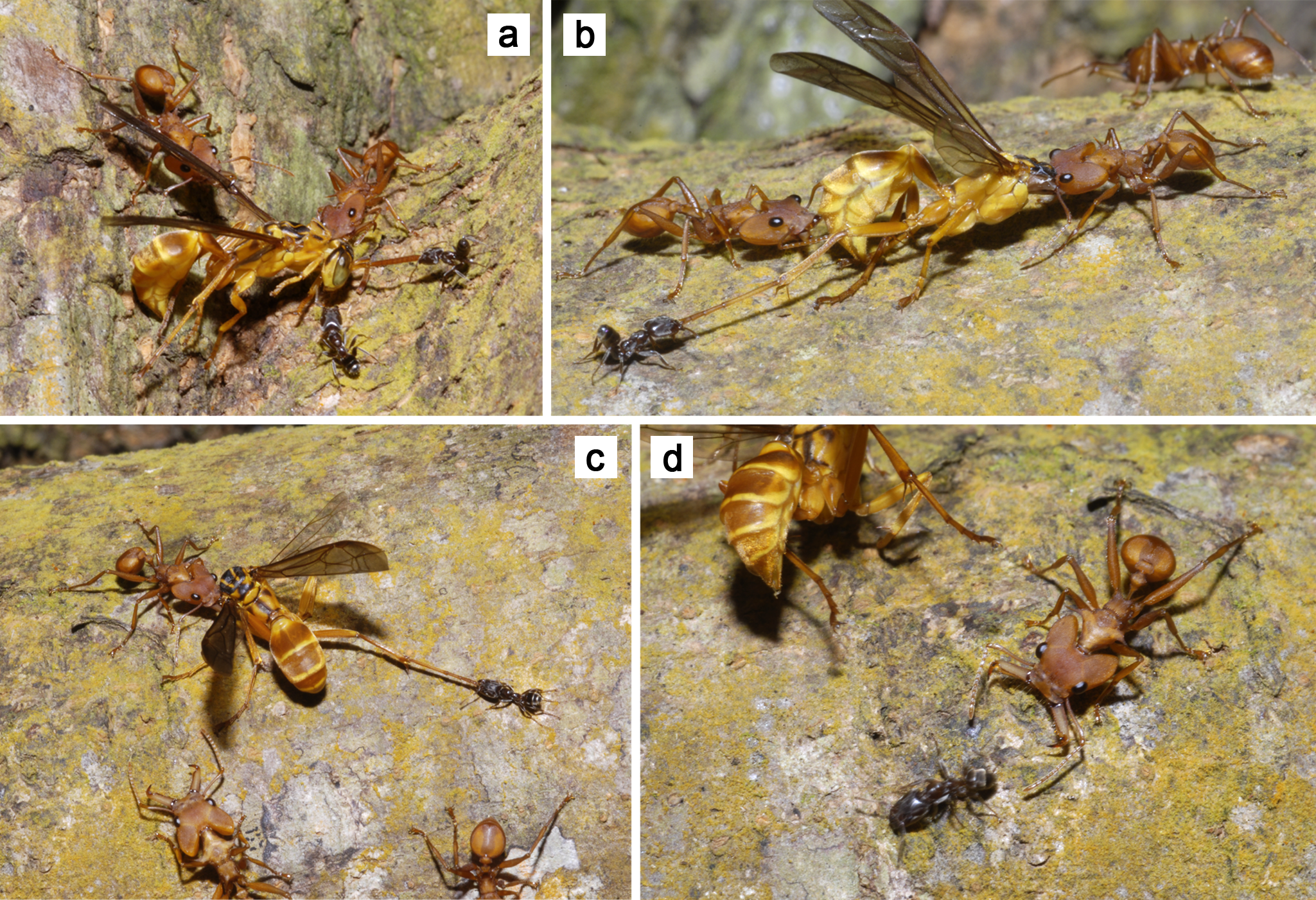

Supplement: Figure S2 — Azteca sp. workers trying to rob a wasp captured by Daceton armigerum workers. (TIF) [file pone.0037683.s004.tif]

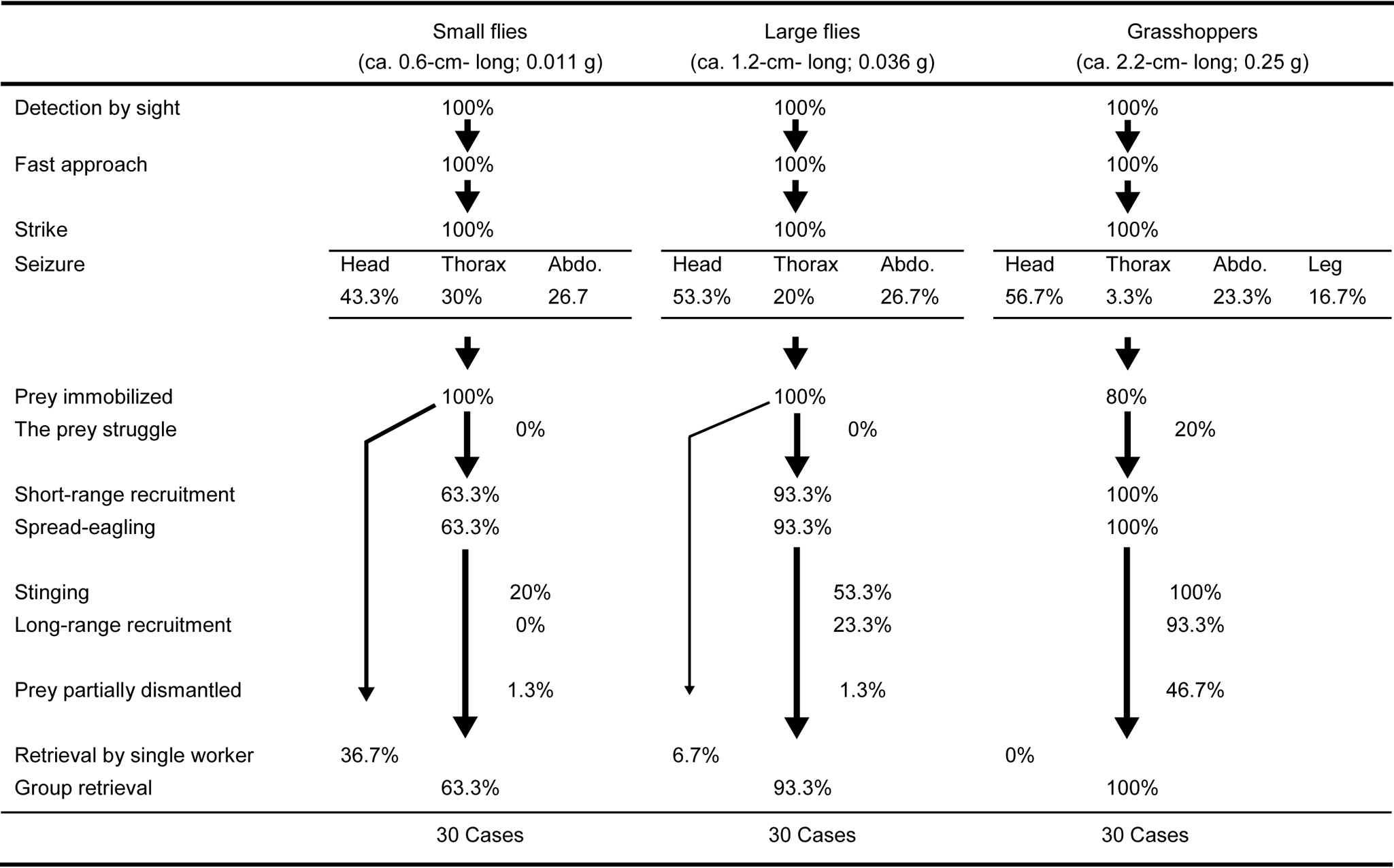

Supplement: Figure S3 — Behavioral sequences during predation by ambushing Daceton armigerum workers when prey land (flies) or are dropped (grasshoppers) less than 3 cm from them. (TIF) [file pone.0037683.s005.tif]

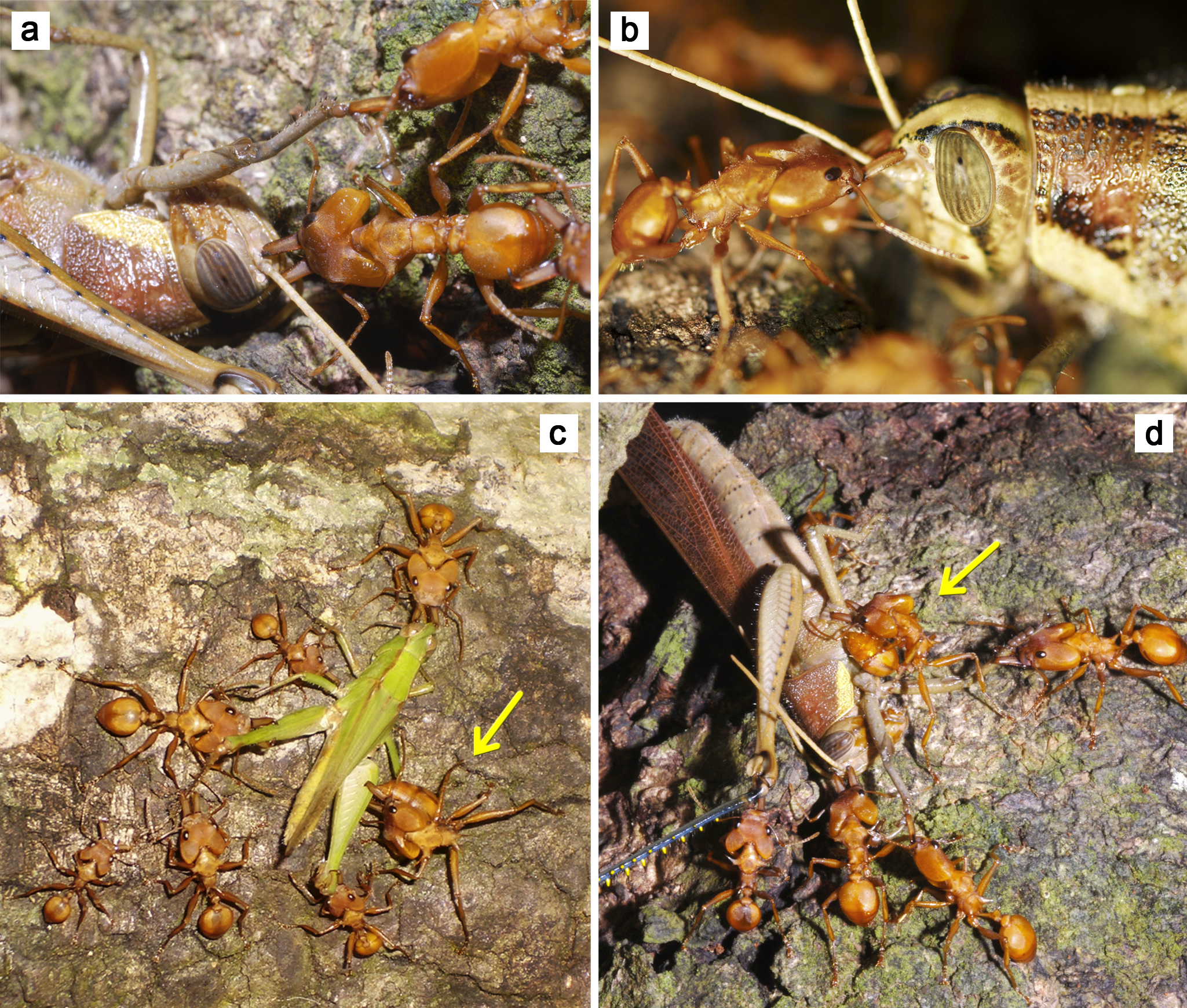

Supplement: Figure S4 — During the attacks ambushing workers face the prey and strike them on the head. This likely numbs the prey until nestmates can be recruited at short range. (TIF) [file pone.0037683.s006.tif]

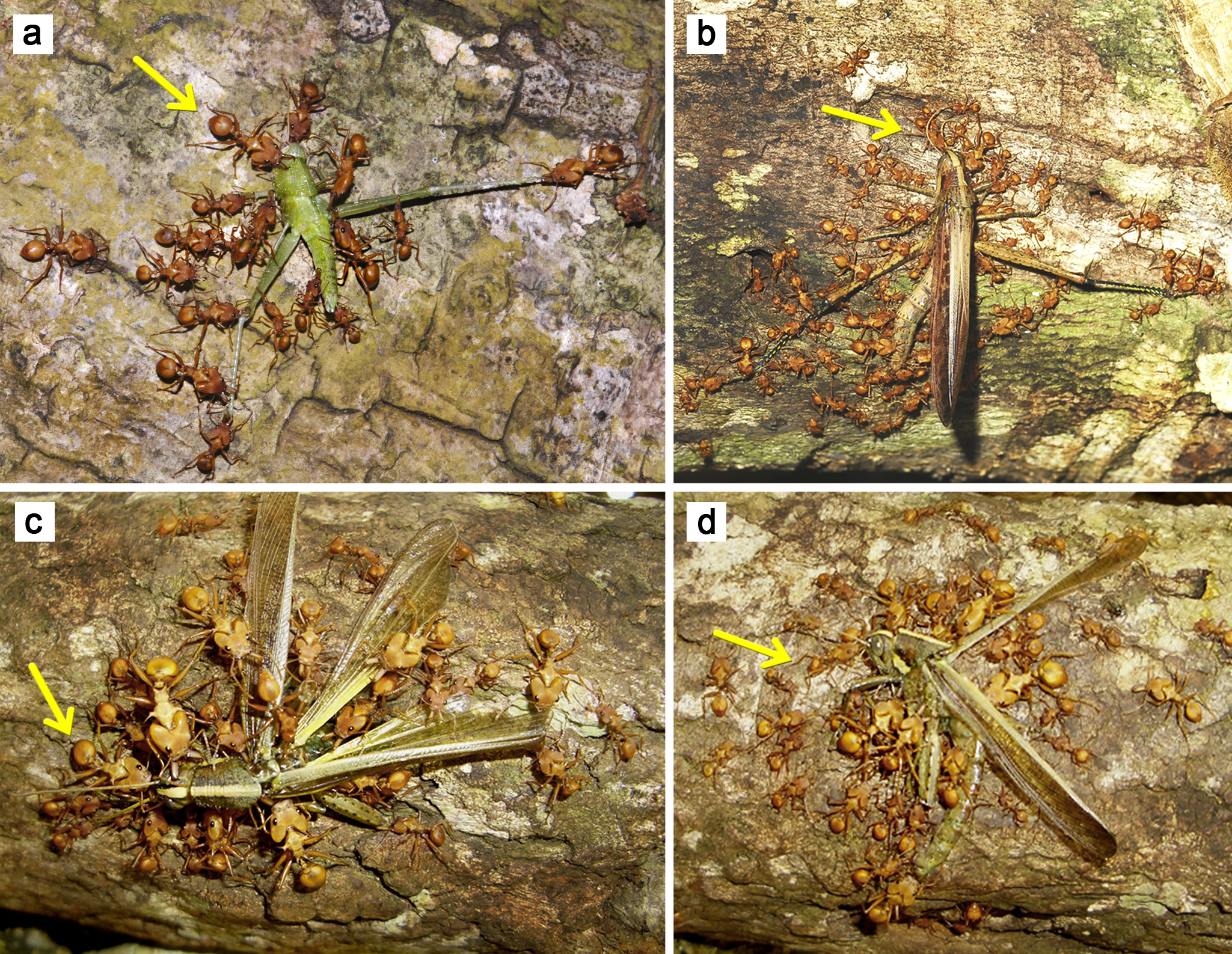

Supplement: Figure S5 — Spread-eagling the prey. (TIF) [file pone.0037683.s007.tif]

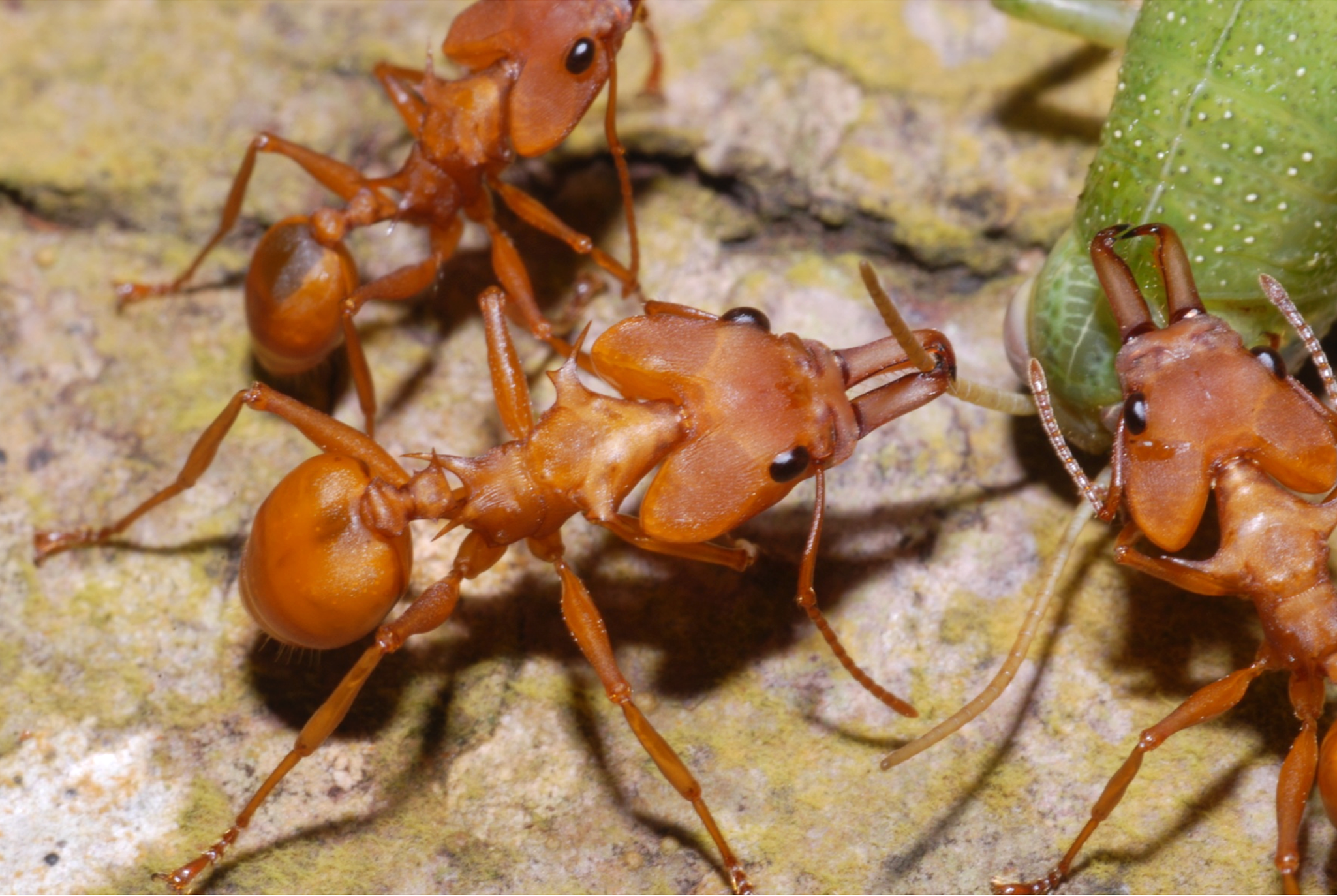

Supplement: Figure S6 — Illustration that the shape of the tip of the Daceton armigerum mandibles permits them to easily seize prey appendages. (TIF) [file pone.0037683.s008.tif]

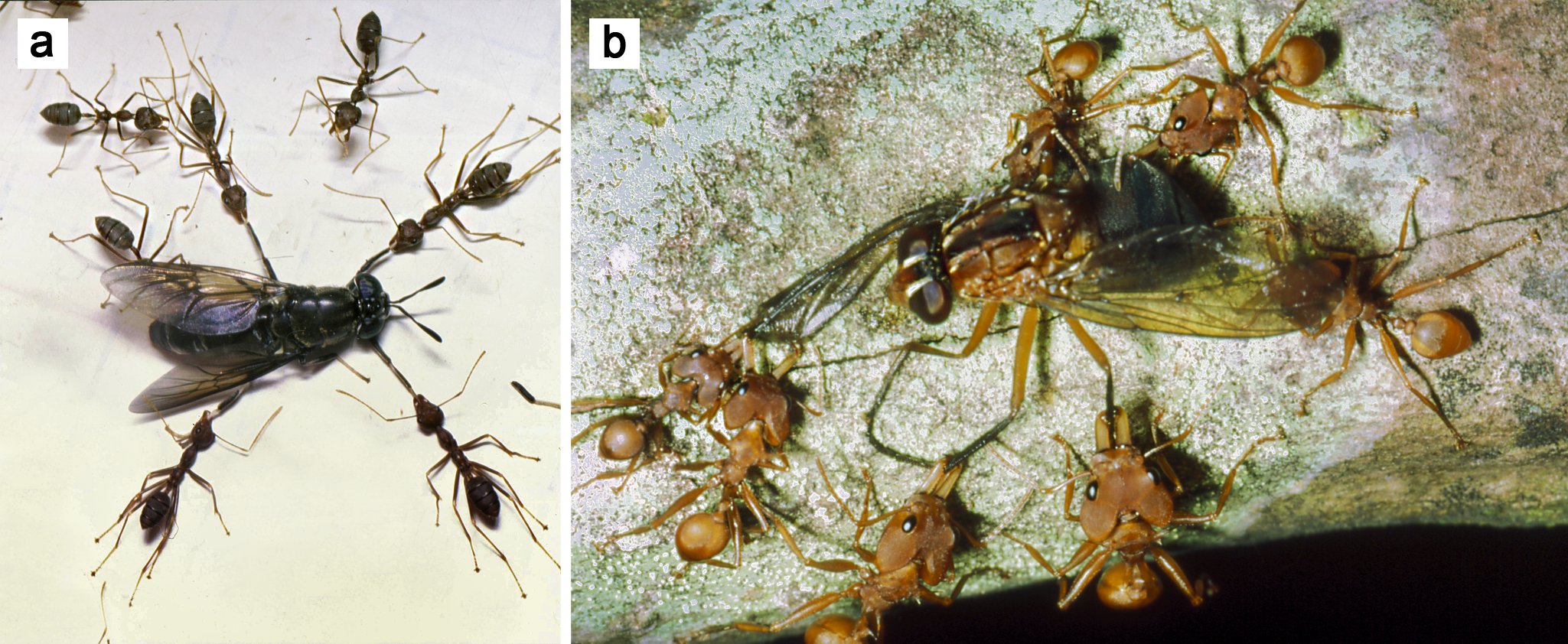

Supplement: Figure S7 — Spread-eagling flies or relatively small prey. (TIF) [file pone.0037683.s009.tif]
